# Influenza as the Predominant Cause of Severe Hepatic Involvement in Children Hospitalized with Acute Respiratory Infections: A Post-COVID-19 Era Analysis

**DOI:** 10.3390/v18070691

**Published:** 2026-06-23

**Authors:** Ozlem Kalaycik Sengul, Suleyman Zahid Akyuz, Ilke Aktas, Ezgi Dilan Sencan, Asude Sule Arikan, Sevliya Ocal Demir, Sebahat Cam

**Affiliations:** 1 Division of Pediatric Gastroenterology, Hepatology and Nutrition, School of Medicine, Istanbul Medeniyet University, 34729 Istanbul, Turkey; dr.ilkeaktas2@gmail.com (I.A.); yukselezgidilan@gmail.com (E.D.S.); suleballi93@gmail.com (A.S.A.); imamoglus@yahoo.com (S.C.); 2 Department of Pediatrics, School of Medicine, Istanbul Medeniyet University, 34700 Istanbul, Turkey; s.zahidakyuz@gmail.com; 3 Department of Pediatric Infectious Diseases, Istanbul Goztepe Prof. Dr. Suleyman Yalcin City Hospital, 34722 Istanbul, Turkey; sevliyademir@gmail.com

**Keywords:** influenza, acute hepatitis, liver enzymes, pediatric infection, respiratory viruses

## Abstract

**Background**: Following the coronavirus disease 2019 (COVID-19) pandemic, increased reports of severe acute hepatitis of unknown etiology in children have raised concerns about virus-associated liver injury. Acute respiratory tract infections (ARTIs) are a common cause of pediatric hospitalization and may be accompanied by reactive hepatitis; however, virus-specific patterns of hepatic involvement remain incompletely defined. This study aimed to evaluate liver involvement associated with ARTIs in hospitalized children. **Methods**: This retrospective study included pediatric patients (<18 years) hospitalized with ARTIs between October 2021 and May 2023. Respiratory viruses were identified via multiplex real-time polymerase chain reaction assays. Liver function tests were systematically evaluated during hospitalization. Transaminase elevations were categorized according to the upper limit of normal (ULN = 40 U/L). Acute hepatic failure was defined according to the Pediatric Acute Liver Failure criteria. **Results**: A total of 179 patients were analyzed (median age: 38 months; 59.2% male). Elevated AST and ALT levels were observed in 24.0% and 8.4% of patients, respectively. Adenovirus was the most frequently detected virus (11.2%), followed by influenza A (7.3%) and parainfluenza virus (6.7%). Severe transaminase elevations (>5 × ULN and >500 U/L) were observed in patients with influenza infection. All cases of acute hepatic failure (*n* = 3) were associated with influenza infection. Other respiratory viruses were associated with mild or transient liver enzyme abnormalities. **Conclusions**: Severe hepatic involvement—including severe transaminase elevation and acute hepatic failure—occurred exclusively in children with influenza infection, particularly influenza B, while mild and transient liver enzyme abnormalities were common across other respiratory viral infections. These findings highlight the importance of targeted liver function monitoring in pediatric influenza patients and provide clinically relevant data on virus-specific hepatic involvement in the post-COVID-19 era.

## 1. Introduction

In late 2021, following the COVID-19 pandemic, a cluster of cases of severe acute hepatitis of unknown etiology (SAHUA) in children was first reported in Alabama, USA [1,2], followed shortly thereafter by similar reports from Scotland [3] in early 2022. These initial observations marked the emergence of a concerning clinical entity characterized by acute liver injury in previously healthy children, in whom infection with classical hepatotropic viruses (hepatitis A–E) could not be identified. Within a short period, case numbers increased substantially across multiple countries, prompting significant international concern [4]. In response to this unexpected surge, the World Health Organization (WHO) issued a global outbreak alert, underscoring the urgent need to clarify the underlying etiology [5].

By early July 2022, 1010 probable cases of SAHUA had been reported from 35 countries in children aged 16 years or younger [5]. Among the pathogens investigated, adenovirus is the most frequently detected; however, its causal role in the development of severe hepatitis remains uncertain [5,6]. Adenovirus infections were well documented prior to the COVID-19 pandemic without being associated with a comparable surge in severe hepatic disease, suggesting that additional factors may be required to trigger clinically severe hepatic involvement [7]. These factors may include viral coinfections, host susceptibility, or immune-mediated mechanisms [8].

Acute respiratory tract infections (ARTIs) represent one of the most common causes of pediatric hospitalization, accounting for approximately 30% of all hospital admissions in children. Viral pathogens are responsible for more than 60% of ARTIs and are a well-recognized cause of nonspecific reactive hepatitis, which often manifests as transient elevations in liver enzymes [9]. The introduction of multiplex real-time polymerase chain reaction (RT–PCR) assays has enabled more accurate identification of respiratory viruses and facilitated detailed investigations into virus-specific clinical manifestations, including extrapulmonary involvement, such as liver enzyme abnormalities.

The COVID-19 pandemic profoundly disrupted the epidemiological landscape of common childhood infections. Non-pharmaceutical interventions—including school closures, physical distancing, and mask use—substantially curtailed the circulation of endemic respiratory viruses, resulting in a period of reduced immunological priming in young children [10]. Following the relaxation of these measures, an atypical and pronounced resurgence of multiple respiratory viruses was observed globally, disproportionately affecting younger, immunologically naïve children who lacked prior exposure to seasonal pathogens [10,11]. This altered immune landscape coincided temporally with the global emergence of unexplained severe acute hepatitis in children, providing a compelling rationale for systematically examining virus-specific patterns of hepatic involvement in the post-pandemic period. Importantly, the temporal overlap between the post-COVID-19 resurgence of respiratory viruses and the unexplained hepatitis outbreak underscores the need for prospective data on extrapulmonary manifestations of respiratory viral infections during this unique epidemiological window. In this context, our institution observed a substantial increase in pediatric hospitalizations due to ARTIs in the post-COVID-19 period, providing an opportunity to address this critical knowledge gap. The aim of this study was to evaluate liver function tests in hospitalized patients with acute respiratory tract infections in the post-COVID-19 era to determine the potential risk of virus-specific acute hepatitis and acute liver failure.

## 2. Materials and Methods

### 2.1. Ethics Statement

This study was approved by the Goztepe Prof. Dr. Suleyman Yalcin City Hospital Ethics Committee (Decision No.: 2023/0954, date: 20 December 2023), and all procedures were performed in accordance with the principles of the Declaration of Helsinki.

### 2.2. Study Design and Population

This retrospective study was conducted on pediatric patients (<18 years of age) who were hospitalized with acute respiratory tract infections in the Department of Pediatric Infectious Diseases between October 2021 and May 2023. All the children underwent 11 respiratory pathogen tests via RT–PCR within the first 48 h of admission (Molecular diagnosis of respiratory tract infections was performed using the Bio-Speedy Respiratory Tract RT-qPCR MX-24S Panel (Bioeksen R&D Technologies, Istanbul, Turkey) on a CFX96 Real-Time PCR System (Bio-Rad, Hercules, CA, USA).

Patient demographics, underlying diseases, medication history, complaints, and laboratory results were recorded at the time of administration. Patients with a history of chronic liver disease and suspected drug toxicity were excluded. Informed consent for participation was not required due to the retrospective nature of the study and the use of anonymized clinical data, in accordance with institutional requirements and ethics committee approval. Patients with incomplete medical records or laboratory data were excluded from the study.

### 2.3. Definitions and Laboratory Investigations

To determine the etiology, blood samples for viral hepatitis (HBV, HCV, HAV, EBV, and CMV) were obtained from patients with acute hepatitis. The patients in the study underwent a pediatric gastroenterology evaluation on the basis of their clinical characteristics and follow-up results. As a result, non-infectious causes of acute hepatitis were assessed in accordance with the European Society for Pediatric Gastroenterology, Hepatology, and Nutrition guidelines [9].

Blood culture and nasopharyngeal swab samples were collected from all patients within the first 48 h of admission to identify etiological infectious agents.

We collected initial aspartate aminotransferase (AST), alanine aminotransferase (ALT), alkaline phosphatase (ALP), gamma-glutamyl transferase (GGT), total bilirubin (TB), direct bilirubin (DB), and albumin levels; the prothrombin time/international normalized ratio (PT/INR); and complete blood counts. We also collected the peak and final AST and ALT levels.

Liver enzyme elevation was defined as AST or ALT values > 40 U/L on the basis of the maximum values observed during follow-up. Acute hepatitis was defined as elevated (>500 U/L) AST or ALT [12]. Transaminase elevations were further stratified by severity as mild (>1–2 × ULN), moderate (>2–5 × ULN), and severe (>5 × ULN and/or an absolute value > 500 U/L). The terms “severe hepatic involvement” and “severe transaminase elevation” are used interchangeably throughout the manuscript to denote this severe category. On the basis of diagnostic investigations, clinicians have determined whether acute hepatitis is of unknown cause. Patients whose cause was confirmed by a clinician (e.g., hepatitis A–E, drug-induced liver injury, or definitive autoimmune hepatitis) were excluded from the study.

Pediatric acute liver failure is defined as biochemical evidence of acute liver injury with an international normalized ratio (INR) ≥ 1.5, accompanied by hepatic encephalopathy or an international normalized ratio (INR) ≥ 2.0, regardless of encephalopathy in a patient without preexisting liver disease within the previous 8 weeks and without correction after parenteral vitamin K administration [13].

### 2.4. Statistical Analysis

Statistical analyses were performed via SPSS version 27.0. Continuous variables were assessed for normality via visual (histograms and Q–Q plots) and analytical methods. As most continuous variables were nonnormally distributed, the data are expressed as medians and interquartile ranges (IQRs). Categorical variables are presented as numbers and percentages.

Comparisons between two independent groups were performed via the Mann–Whitney U test for continuous variables and the chi-square test or Fisher’s exact test, as appropriate, for categorical variables. Comparisons among more than two groups were conducted via the Kruskal–Wallis test. Post hoc analyses were not performed because of the limited number of patients in certain subgroups. Viral subgroup analyses were conducted by comparing virus-positive patients with those negative for the respective viruses. All the statistical tests were two-sided, and a *p*-value of <0.05 was considered to indicate statistical significance.

## 3. Results

During the study period, 179 pediatric patients hospitalized with acute respiratory tract infections were included in the analysis. The median age was 38 months (IQR, 12–73.5), and 106 patients (59.2%) were male. The median length of hospital stay was 6 days (IQR, 4–8). The baseline laboratory findings of the patients are summarized in Table 1.

The median length of hospital stay did not differ significantly between patients with a single viral infection and those with multiple viral infections (7 days [IQR, 4–8] vs. 6 days [IQR, 4.25–7], *p* = 0.321). Similarly, no significant difference in the length of hospital stay was observed between patients with and without ALT elevation (median 6 days in both groups, *p* = 0.58).

Elevated ALT levels (>40 U/L) were detected in 8.4% of patients, whereas AST elevation was observed in 24% of patients. Concomitant elevation of both enzymes occurred in 7.8% of the patients, indicating that AST elevation was more prevalent than ALT elevation in the overall cohort.

Adenoviruses were the most frequently detected viral pathogens (11.2%), followed by influenza A (7.3%) and parainfluenza virus (6.7%). A single viral agent was observed in 33% of patients, whereas 5% had multiple viral infections.

The transaminase content varies according to the viral pathogen. Severe transaminase elevations were observed predominantly in patients with influenza B infection, whereas other viral infections were associated with relatively lower enzyme levels. When elevation was defined as >40 U/L, influenza B infection was associated with the highest proportion of elevated AST (80%) and ALT (60%) levels. Elevated AST levels were also frequently observed in RSV (50%) and parainfluenza virus infections (41.7%). No ALT elevation was detected in adenovirus-positive patients, whereas AST elevation was detected in 35% of patients.

When maximum transaminase values during follow-up were analyzed, maximum AST elevations (>40 U/L) were most frequently observed in patients with influenza B (80%), RSV (50%), and parainfluenza virus infection (41.7%). Maximum ALT elevations (>40 U/L) were predominantly detected in influenza B (60%) and influenza A (15.4%) infections, whereas such elevations were uncommon or absent in other viral pathogens.

**Figure 1:**
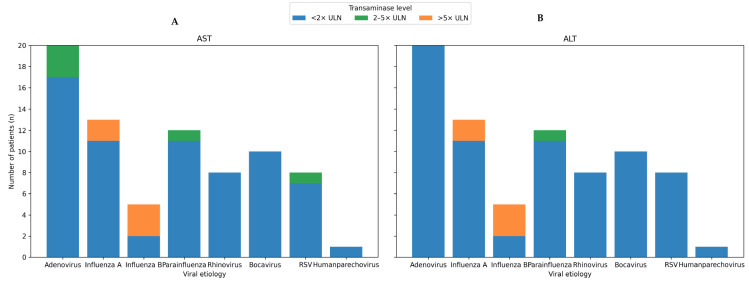
Distribution of maximum AST and ALT levels according to viral etiology: (**A**) Maximum AST levels. (**B**) Maximum ALT levels. The transaminase levels were classified according to the maximum recorded value during the clinical course as <2 × upper limit of normal (ULN), 2–5 × ULN, or >5 × ULN. The bars represent the absolute number of patients.

The distributions of the maximum ALT and AST levels according to viral pathogens are presented in Figure 1A and Figure 1B, respectively. Most patients presented with transaminase levels within ≤2 × the upper limit of normal (ULN). Severe elevations (>5 × ULN) were observed in patients with influenza virus infection.

Severe transaminase elevations (AST or ALT > 500 U/L) were observed only in influenza infections, occurring in 15.4% of influenza A and 40% (AST)/60% (ALT) of influenza B cases, with no such cases in other viral infections.

To further characterize virus-specific differences in hepatic involvement, maximum AST and ALT levels were compared between virus-positive and virus-negative patients for each detected pathogen using the Mann–Whitney U test (Table 2). Influenza B infection was associated with significantly higher maximum AST (*p* = 0.008) and ALT (*p* = 0.014) levels than influenza B-negative status. No other pathogen showed a statistically significant association with maximum AST or ALT (all *p* > 0.05).

Five patients were diagnosed with influenza B infection; this subgroup exhibited severely elevated transaminase levels, with median maximum AST and ALT values of 369 U/L and 727 U/L, respectively.

Acute hepatic failure was identified in three patients, all of whom had influenza virus infection (two with influenza A infection and one with influenza B infection). None of the patients with other viral infections developed hepatic failure.

## 4. Discussion

In this retrospective study, we evaluated liver enzyme abnormalities in pediatric patients hospitalized with acute respiratory tract infections during the post-COVID-19 period. Our findings demonstrate that while mild elevations in transaminase levels are relatively common, clinically severe hepatic involvement, particularly in influenza B, is rare and occurs predominantly in association with influenza virus infection. Notably, severe transaminase elevation and acute hepatic failure are observed exclusively in patients with influenza infection, highlighting a virus-specific risk for significant hepatic involvement.

Consistent with previous reports, AST elevation was more frequently observed than ALT elevation in the overall cohort [9,14]. This pattern suggests that transaminase abnormalities associated with respiratory viral infections are often mild and may reflect systemic inflammation or extrahepatic sources rather than primary hepatocellular injury [14]. Indeed, adenoviruses, respiratory syncytial viruses, parainfluenza viruses, and other nonhepatotropic respiratory viruses are largely associated with AST-predominant or low-grade enzyme elevations, supporting the concept of nonspecific reactive hepatitis in the setting of acute infection.

In contrast, influenza B infection was associated with severely elevated transaminase levels, with the highest proportion of patients exceeding clinically meaningful thresholds, including >5 × the upper limit of normal and >500 U/L. Moreover, all cases of acute hepatic failure, defined by elevated INR values, occurred in patients with influenza infection, including one patient with influenza B. Although the number of influenza B cases was limited, the consistency and severity of liver enzyme abnormalities observed in this subgroup suggest a distinct clinical pattern characterized by more pronounced hepatic involvement.

The association between influenza infection and liver injury has been previously described, with proposed mechanisms including cytokine-mediated immune injury, hypoxic hepatitis secondary to systemic illness, and possible direct viral effects on hepatocytes [15]. Influenza B, in particular, has been reported in isolated case series to cause severe hepatitis and acute liver failure in children [16]. Experimental data support a predominantly indirect, immune-mediated mechanism of influenza-associated hepatic injury. In a murine model, influenza A virus infection induced hepatocellular injury and aminotransferase elevation in the absence of detectable viral replication within the liver, with activation of hepatic Kupffer cells and hepatocyte apoptosis implicated as major contributors to liver injury [17]. More broadly, mechanistic studies of pediatric acute liver failure across diverse etiologies highlight the roles of innate immune activation, hepatocyte apoptosis, and impaired hepatic regeneration rather than direct viral cytopathic effects [18]. These findings are consistent with our observation that, despite severely elevated transaminase levels, influenza-associated hepatic injury in our cohort was transient and self-limited, supporting an immune-mediated and potentially reversible process rather than progressive hepatocellular destruction. In our cohort, severe transaminase elevation and pediatric acute liver failure occurred exclusively in patients with influenza infection, emphasizing the importance of careful biochemical monitoring and timely recognition of severe hepatic involvement in children with influenza.

Importantly, despite severely elevated transaminase levels, none of the influenza B-infected patients in our cohort progressed to irreversible liver failure or required liver transplantation. Liver enzyme abnormalities were transient and improved during follow-up, suggesting a generally favorable clinical course when appropriate supportive care is provided. This observation underscores the importance of distinguishing transient virus-associated hepatitis from progressive liver disease, thereby avoiding unnecessary invasive investigations in clinically stable patients.

In contrast to global observations during the outbreak of severe acute hepatitis of unknown etiology [1,3,4], adenovirus infection in our cohort was not associated with significant ALT elevation, severe transaminase abnormalities, or acute hepatic failure. These findings support the hypothesis that adenovirus detection alone may be insufficient to explain severe hepatic injury and that host factors, immune dysregulation, or specific viral subtypes may be necessary contributors to hepatitis.

Furthermore, our findings provide an additional perspective on the global SAHUA outbreak. When applying the WHO probable case definition for severe acute hepatitis of unknown etiology in children (AST or ALT > 500 U/L in a child ≤ 16 years of age with exclusion of hepatitis A–E) [5] to our cohort, six children with severe influenza-associated hepatitis (three with influenza A and three with influenza B) would have fulfilled these surveillance criteria had the underlying influenza infection not been identified through systematic multiplex respiratory RT-PCR testing. Among these patients, three developed pediatric acute liver failure (two influenza A and one influenza B), and all had negative investigations for hepatitis A–E as well as EBV and CMV infections. This observation suggests that severe influenza-associated hepatitis may represent an underrecognized contributor to reported SAHUA case clusters in settings where comprehensive respiratory viral testing is not routinely performed and supports the inclusion of respiratory viral testing, including influenza, in the diagnostic evaluation of children presenting with severe acute hepatitis of initially unknown cause.

This study has several limitations. Its retrospective design and single-center setting may limit generalizability. The number of patients in certain viral subgroups, particularly influenza B, was small, limiting the statistical power. Nevertheless, the consistency of findings across multiple severity thresholds strengthens the validity of our conclusions. In addition, beyond the multiplex respiratory viral panel and serological exclusion of classical hepatotropic viruses, further investigations such as adenovirus subtyping and metagenomic sequencing were not performed.

## 5. Conclusions

The principal finding of this study is that severe hepatic involvement—defined as transaminase elevation >5 × ULN or >500 U/L and/or acute hepatic failure—occurred exclusively among children with influenza infection, particularly influenza B, despite influenza accounting for a minority of the overall cohort. Mild transaminase elevation is common in children hospitalized with acute respiratory tract infections, whereas severe hepatic involvement is rare. In contrast, adenovirus infection and infection with other respiratory viruses are largely associated with mild or transient liver enzyme abnormalities. These findings highlight the importance of virus-specific risk assessment and targeted liver function monitoring, especially in pediatric patients with influenza infection, and contribute to a more precise understanding of hepatic involvement in respiratory viral infections in the post-COVID-19 era.

## Figures and Tables

**Table 1:** Characteristics and baseline laboratory findings of the patients.

Characteristics	Number (%)	Median	Interquartile Range (IQR)
Sex			
Male	106 (59.2)	106 (59.2)	
Female	73 (40.8)		
Median age (Month)		38	12–73.5
Initial laboratory data			
AST, U/L	179	31	24.5–40
ALT, U/L	179	14	10–22
Albumin, gr/dL	179	4.1	3.8–4.4
GGT, IU/L	141	12	9–28
ALP, U/L	143	163	134.5–218.5
T. Bil, mg/dL	151	0.26	0.17–0.43
D. Bil, mg/dL	151	0.14	0.10–0.29
WBC	179	11,300	7900–16,000
Hb	179	11.5	10.8–12.5
PLT	179	302,000	234,000–392,000
PT/INR	31	15.35/1.1	13.9–16.8/1–1.33
CRP	179	20.6	5.49–68.6
Peak laboratory data			
AST, U/L	179	31	25–41
ALT, U/L	179	14	10–25.5
INR	31	1.1	1–1.33
Final laboratory data			
AST, U/L	179	40	32–51.8
ALT, U/L	179	42	21.3–68.3

**Table 2:** Comparison of maximum AST and ALT levels between pathogen-positive and pathogen-negative patients (Mann–Whitney U test).

Pathogen	Positive, *n* (%)	Max. AST, Positive (U/L), Median (IQR)	Max. AST, Negative (U/L), Median (IQR)	*p*-Value (AST)	Max. ALT, Positive (U/L), Median (IQR)	Max. ALT, Negative (U/L), Median (IQR)	*p*-Value (ALT)
Adenovirus	20 (11.2)	29 (23–56)	31 (25–40)	0.998	13 (10–18)	15 (10–26)	0.199
Influenza A	13 (7.3)	31 (25–38)	31 (25–41)	0.996	13 (12–16)	15 (10–24)	0.881
Influenza B	5 (2.8)	369 (60–1416)	31 (24–40)	0.008 *	727 (20–1413)	14 (10–22)	0.014 *
Rhinovirus	8 (4.5)	28 (18–31)	32 (25–41)	0.067	12 (9–14)	15 (10–25)	0.211
Bocavirus	10 (5.6)	32 (30–36)	31 (24–41)	0.615	12 (11–15)	15 (10–25)	0.477
RSV	8 (4.5)	40 (32–50)	31 (25–40)	0.287	30 (19–38)	14 (10–22)	0.103
Parainfluenza virus	12 (6.7)	38 (32–48)	31 (24–40)	0.144	16 (12–19)	14 (10–25)	0.544
Human parechovirus	1 (0.6)	39 (—)	31 (25–41)	0.462	11 (—)	14 (10–24)	0.498

* *p* < 0.05. Abbreviations: AST, aspartate aminotransferase; ALT, alanine aminotransferase; IQR, interquartile range; RSV, respiratory syncytial virus.

## Data Availability

The original contributions presented in the study are included in the article; further inquiries can be directed to the corresponding author.
